# ScreenGarden: a shinyR application for fast and easy analysis of plate-based high-throughput screens

**DOI:** 10.1186/s12859-022-04586-1

**Published:** 2022-02-05

**Authors:** Cinzia Klemm, Rowan S. M. Howell, Peter H. Thorpe

**Affiliations:** 1 School of Biological and Behavioural Science, Queen Mary University of London, Mile End Road, London, E1 4NS UK; 2 Present Address: UCL Cancer Institute, University College London, 72 Huntley Street, London, WC1E 6DD UK

**Keywords:** Genomics, Yeast, Automated data analysis

## Abstract

**Background:**

Colony growth on solid media is a simple and effective measure for high-throughput genomic experiments such as yeast two-hybrid, synthetic dosage lethality and Synthetic Physical Interaction screens. The development of robotic pinning tools has facilitated the experimental design of these assays, and different imaging software can be used to automatically measure colony sizes on plates. However, comparison to control plates and statistical data analysis is often laborious and pinning issues or plate specific growth effects can lead to the detection of false-positive growth defects.

**Results:**

We have developed ScreenGarden, a shinyR application, to enable easy, quick and robust data analysis of plate-based high throughput assays. The code allows comparisons of different formats of data and different sized arrays of colonies. A comparison of ScreenGarden with previous analysis tools shows that it performs, at least, equivalently. The software can be run either via a website or offline via the RStudio program; the code is available and can be modified by expert uses to customise the analysis.

**Conclusions:**

ScreenGarden provides a simple, fast and effective tool to analyse colony growth data from genomic experiments.

**Supplementary Information:**

The online version contains supplementary material available at 10.1186/s12859-022-04586-1.

## Background

The budding yeast *Saccharomyces cerevisiae* is a widely used model organism to understand basic molecular processes in eukaryotic cells. Over the past decades, the development of new genetic techniques enabled the creation of comprehensive clone and gene deletion libraries in yeast. These libraries can be used for many different high-throughput experiments, such as synthetic lethality and synthetic dosage lethality screens [1–3], chemical genetic screens [3], and yeast two-hybrid and Synthetic Physical Interaction screens to unravel unknown protein–protein interactions [4, 5]. Although investigating different research aspects, the common read-out of these screening methods is colony growth on solid media. Libraries are typically organised in arrays of 96, 384 and 1536 colonies per plate and the colony size of experimental and control conditions are compared to determine growth effects. Library-based screens efficiently generate robust and large datasets in a short time, however, data analysis can be challenging, and merely visual comparison of colonies lacks normalisation of plate differences and is highly subjective. Quantitative data analysis of colony growth can be used to define the strength of growth defects in an unbiased manner. Colony size can be quantified using tools such as ImageJ, HT Colony Grid Analyser, CellProfiler, gittr or Spotsizer [6–9], but these have limited capacity for downstream analysis. Other tools which allow more sophisticated data analysis include proprietary tools such as PhenoBooth Colony Imager (Singer Instruments Ltd, UK), or the *ScreenMill* software suite. The ‘DR engine’ and ‘SV engine’ of the *ScreenMill* software suite were developed to facilitate statistical analysis and offered web-based applications which allowed reviewing and visualising of screening data [6]. However, *ScreenMill* was designed to compare each experimental plate to a single control. Assays that compare experimental plates to two controls, such as Synthetic Physical Interaction screens, require further data processing, which is laborious and can lead to errors. Furthermore, the *ScreenMill* web application is currently not accessible and the software is composed of different programming languages making it difficult to run the analysis for inexperienced data analysts.

To simplify the analysis of plate-based high throughput screens, we have developed ScreenGarden, a shinyR application [10] for statistical analysis of screen-based assays, which compares colony growth of experimental and control plates independent of *ScreenMill*s’ ‘DR engine’ and ‘SV engine’. ScreenGarden can be run as a web application or offline using RStudio [11], which makes it also very easy to adjust and customise the script. ScreenGarden is further developed to facilitate screen analysis which compare experimental plates to two independent controls. Furthermore, ScreenGarden allows direct quality control of screens and plotting of data without exporting the output files into other data analysis software. At the same time, ScreenGarden produces the raw numbers behind each step of data analysis enabling more sophisticated data interpretation downstream for users who require this.

### Implementation

**Fig. 1:**
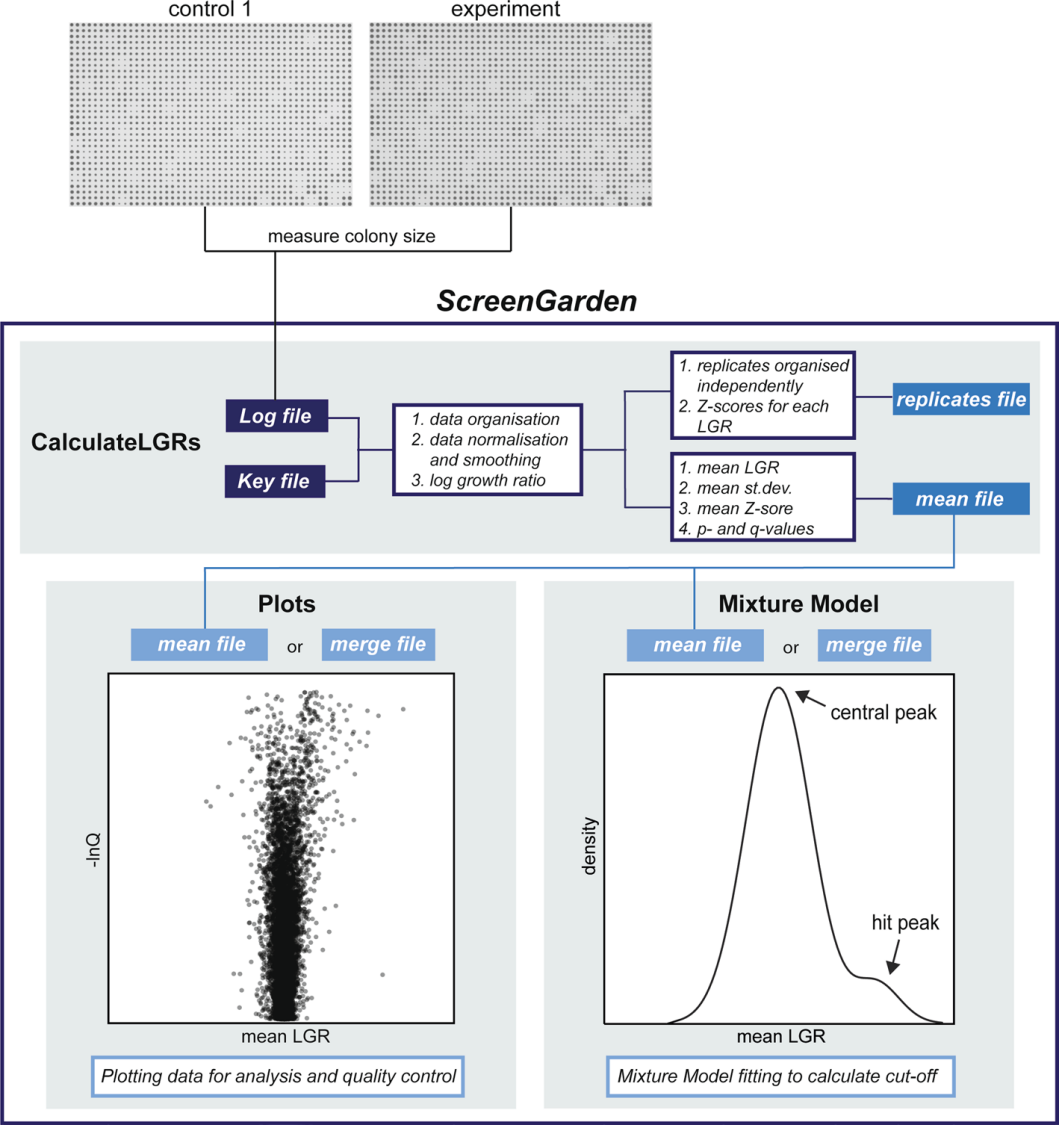
Steps of data analysis using ScreenGarden. The ScreenGarden application offers a tool for stepwise analysis of plate-based high-throughput screens and was specifically adapted to facilitate the analysis of screens with one or two independent controls. The ‘Calculate-LGRs’ script performs statistical analysis of colony sizes compared to a single control. After statistical analysis, the data can be downloaded as a ‘mean file’, which contains the average data of replicates, or as a ‘replicates file’, which contains all data of individual controls. The ‘mean file’, or a ‘merge file’ which is generated by combining the datasets of two independent controls, can be uploaded and plotted for quality control and data visualisation using the ‘Plots’ tool. In addition, the user can choose to define cut-off thresholds using the ‘Mixture Model’ tool

The ScreenGarden application was designed to enable statistical analysis of plate-based assays using colony size as a readout, by comparing colonies from experimental plates to colonies of control plates. The arrangement of strains on these two plates must be identical, i.e., the same strains must be in the same positions on the control and experimental plates. Data analysis using ScreenGarden can be performed in one step if there is a single control condition for each experiment (Fig. 1). Log growth ratios (LGRs) and Z-scores are calculated for a single control using the ‘CalculateLGRs’ command. The user can download a ‘mean file’ which averages the data over the number of replicates, or a ‘replicates file’, which contains the separate data of each individual replicate. Additionally, ScreenGarden can combine data from experiments that use two independent controls using the ‘Combine2controls’ tab; the comparisons to two different controls are combined and downloaded as a ‘merge file’. Finally, the data can be plotted automatically for analysis and quality control, and plots can be directly downloaded from the website. The ScreenGarden app can be run either online (via https://screengarden.shinyapps.io/screengardenapp/) or offline as an R script. The online version provides a straightforward interface with instructions and a short introductory video to provide an example of how to use the software. Users who would prefer to run the script in a local environment can download the R script and run this via an R package, such as Rstudio. Advantages of using the R script offline include the ability to modify the code if required for specialist analysis or to run the software without reliance upon the shinyapp.io web hosting service. A detailed description of how to use the ScreenGarden web application can be found in the appendix (Additional file 1) or downloaded from the ScreenGarden homepage. We have further included a video guide to explain the steps of ScreenGarden analysis and how to download the code, if the user wishes to deploy ScreenGarden offline using RStudio. These video guides can be viewed on the Thorpe lab website: https://www.thorpelab.org/screengarden.

Here, we applied ScreenGarden analysis to previously published data from genome wide Synthetic Physical Interaction screens [12] to compare this software with another established method. Synthetic Physical Interaction screens rely on a GBP-GFP binding system to forcibly associate GBP-tagged proteins to the yeast proteome [5] and a negative impact on cell-growth upon protein–protein interaction is defined as a Synthetic Physical Interaction.

## Results

### Comparison of experimental and control colony sizes

**Fig. 2:**
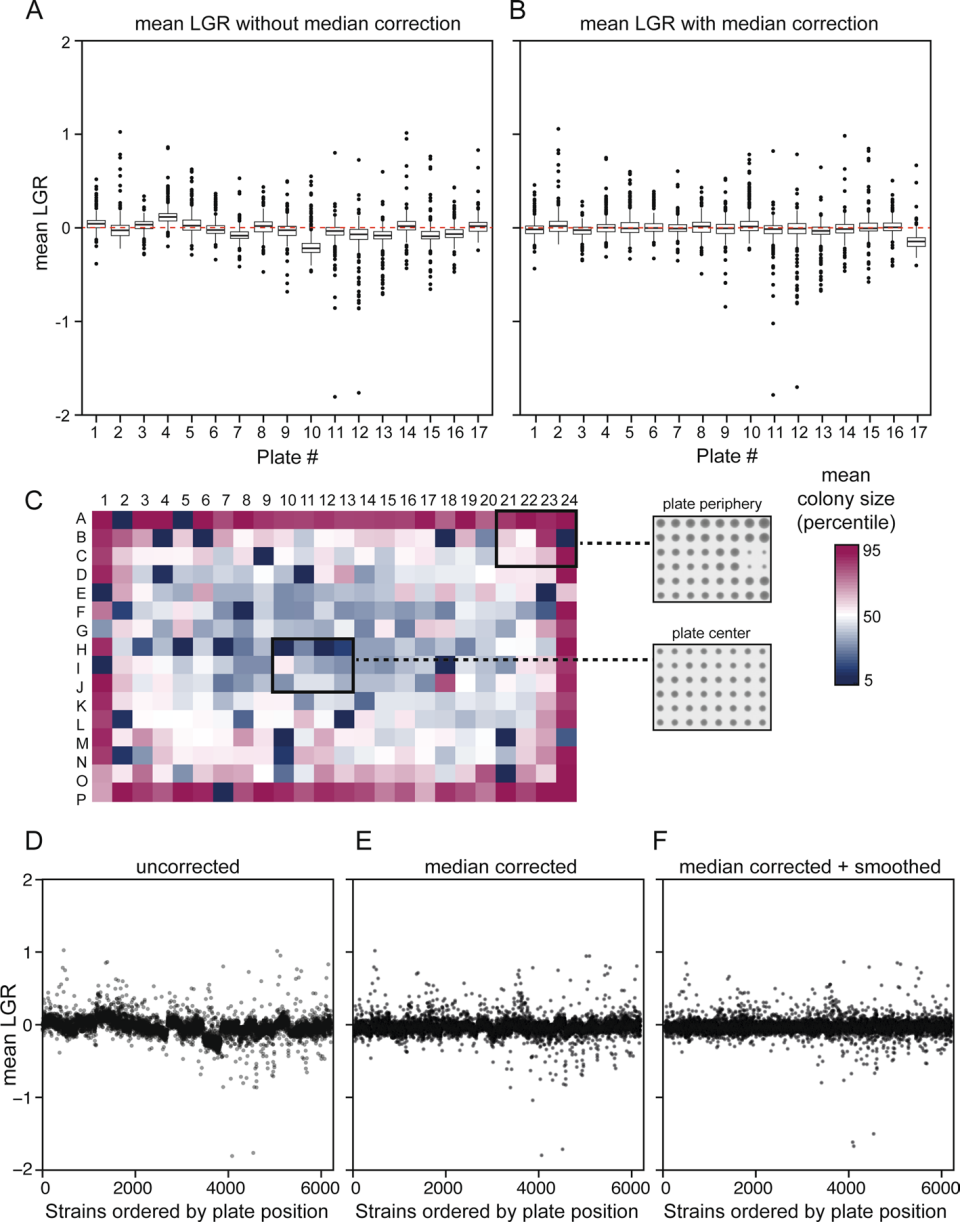
Median correction of colony sizes reduces the influence of plate differences. **A** External factors and discrepancies in pinning can result in differences in colony growth between plates. The mean LGRs organised by plate without median correction are plotted. **B** Correction of colony sizes using the plate median counteracts these plate differences, median correction data from **A** is plotted. **C** A heatmap shows spatial anomalies of colony sizes on plate especially at the plate periphery. Red squares indicate a colony size greater than the plate median and blue squares highlight smaller colonies. The inset shows an example of the raw data. **D**–**F** Mean LGRs are plotted against the yeast library with the data organised on the x-axis, by plate, row and column to highlight the impact of plate differences and spatial anomalies. Including the spatial smoothing algorithm further abolishes any differences based on colony position on plate

ScreenGarden analysis can be performed with different array sizes (384 and 1536 colonies on each plate) and a replicate number of 1, 4 or 16 colonies per yeast library strain. The software requires a ‘Log file’ and a ‘Key file’ as input files. Here, we used *ScreenMills’ CM Engine* to measure colony sizes on plate, which automatically produces a ‘Log file’ as a list of colony sizes ordered by plate position (starting from A1, A2, A3 … H12 for 96 colonies on plate, Additional file 2). Other software tools, such as HT Colony Grid Analyser, can be used to measure colony sizes on plate, but files have to be converted to the specified format (Additional file 3). The ‘Key file’ contains information about the yeast library and assigns the genotype of each strain to its specific plate position (Additional file 4). It is necessary that both, ‘Log file’ and ‘Key file’ are in the format as shown in the examples and that the files are uploaded into ScreenGarden as tab-delimited or comma-separated files. Here, we applied ScreenGarden analysis to Synthetic Physical Interaction screen data with the outer kinetochore subunit Dad2 (Additional file 5). In this screen, GBP-tagged Dad2 was recruited to 6234 different GFP-strains and the screen was performed in 4 replicates, 1536 colonies on a total of 17 plates. First, colony sizes are normalised by the plate median to correct for plate specific effects on growth. Median plate correction is important to prevent false-positive growth defects which might occur due to differences in nutrition, humidity and other external factors [13] (Fig. 2A, B). However, for screens with a high number of growth defects, median-normalisation should be omitted, as a low median colony size might reflect an experimentally valid negative effect on growth. If more than half of the experimental colonies on a plate are affected, the median colony size of the plate will represent the size of affected colonies rather than the ones insensitive to the treatment or condition. Alternatively, the data can be normalised to the median growth value of positive control colonies located at specific positions on the plate which have to be identified as ‘Control’ in the identifier (ID) column in the ‘Key file’. A second difficulty for plate-based screens are spatial anomalies within a plate array. Colonies often grow faster at the plate periphery (Fig. 2C) as there is less competition for nutrients [14, 15]. We have incorporated a simple smoothing algorithm from Ólafsson and Thorpe [5], which adjusts colony sizes based on their plate position to counteract spatial anomalies across the plate. Incorporating the smoothing algorithm into ScreenGarden analysis successfully limits spatial effects (Fig. 2F). Smoothing is optional and, as for median-corrected plate normalisation, should only be selected if most colonies are not affected in growth on experimental plates. After median-correction and smoothing, LGRs are calculated separately for each replicate of each strain on each plate. The LGR is the natural logarithm of the ratio of the control colony size divided by the experimental colony size. The difference between the control and experimental replicates is evaluated by applying a Student’s t-Test to generate a p-value for each comparison. To compensate for false positive growth defects, which naturally occur in large-scale screens, these p-values are adjusted using a false discovery rate (FDR) correction method after Benjamini and Hochberg [16], resulting in more conservative q-values. For both, p-values and q-values, the negative natural logarithm is determined, which is useful for generating Volcano plots which compare LGRs against their p- or q-values. Subsequently, mean LGRs are determined as the average of the 4 or 16 replicate LGRs. Finally, Z-scores for each mean LGR are calculated, which can be used to assess growth defects.

The ‘CalculateLGRs’ tool produces two output files, a ‘replicates file’ where each replicate is listed independently, and a ‘mean file’ which contains the averaged LGRs and Z-scores of the replicates combined. Both files can be downloaded directly from the website as ‘.csv’ files and easily imported into R, Excel and other applications for data analysis.

### Combining two independent controls

**Fig. 3:**
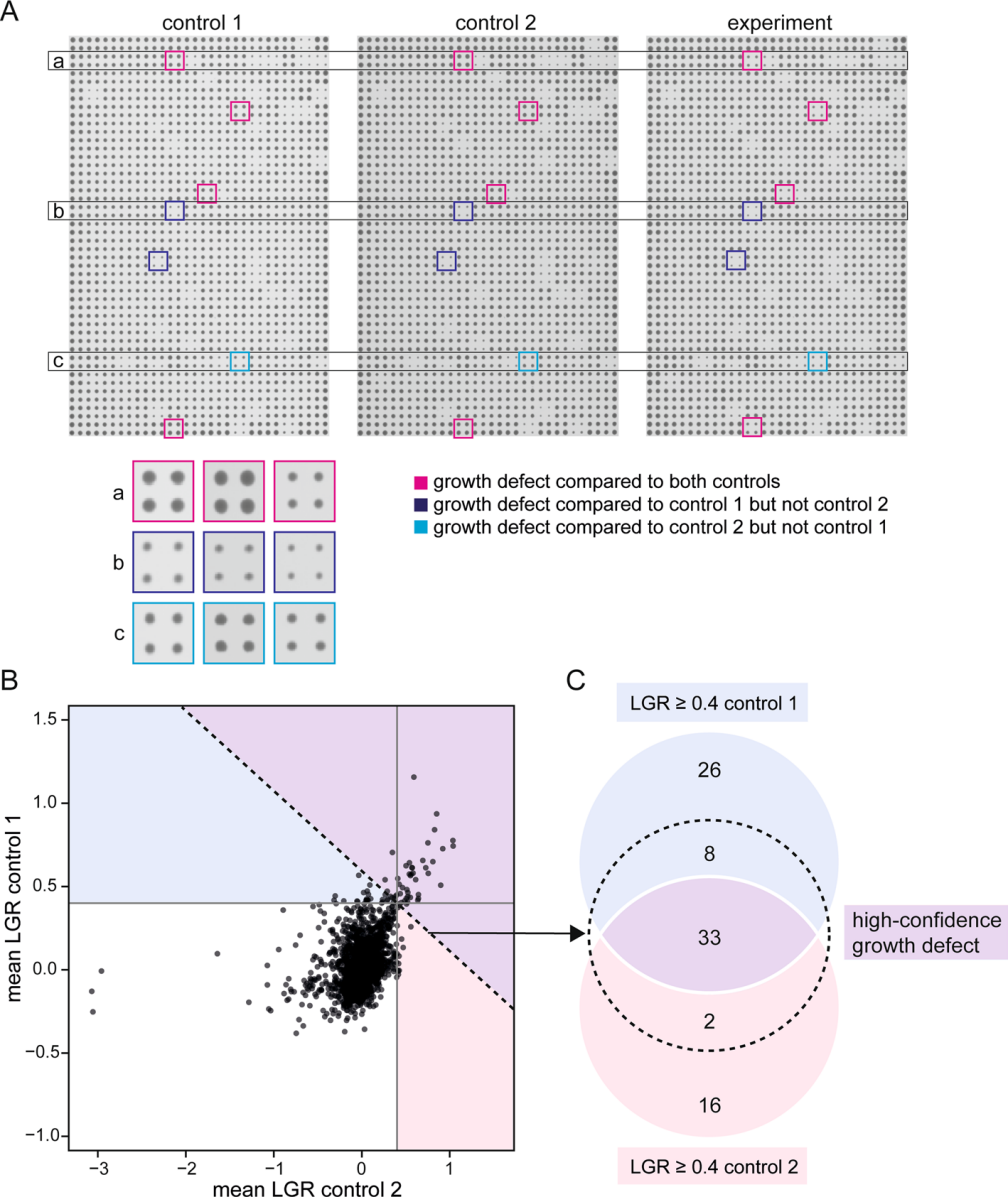
Combining analyses with two independent controls using ScreenGarden. **A** The raw data (plate images) from two control plates to the same experimental plate are shown together for one 1536 density plate with four replicates per strain. Control-specific hits are highlighted in red (control 1) and blue (control 2) boxes respectively. **B** The LGR values for comparing the experiment independently with each control are plotted. The dashed line visualises the empirical cut-off value of LGR ≥ 0.4 for the average LGRs of both control comparisons. **C** These data are shown as a Venn diagram, with the dashed circle indicating all data with an average LGR (of the two controls) ≥ 0.4. Using two controls rather than one defines a subset of high-confidence growth defects and excludes control-specific effects

The second, optional step of screen analysis using ScreenGarden is the ‘Combine2controls’ tool, which is designed for plate-based screens with two independent controls (Fig. 3A). After separately running the ‘Calculate LGRs’ script with each control, the resulting two ‘mean files’ can be uploaded and joined to a single ‘merge file’. The ‘merge file’ includes all the information from the single control analyses and further includes the mean LGRs and Z-scores and the maximum of p- and q-values from both controls. We chose the maximum p or q-value from both independent control datasets as a measure for significance rather than calculating combined p- and q-values using Fisher’s method [17], since the data originates from a single experimental dataset with different controls and the two p- and q-values are not truly independent. The maximum q-value should not be considered a measure of statistical likelihood for mean LGRs of two controls, but rather facilitate the identification of false positive growth defects based on pinning errors. The ‘Plots’ function of ScreenGarden allows these data to be compared, for example to compare the LGR values produced by the two controls. For example, for a Dad2 Synthetic Physical Interaction screen dataset, 18.2% (control 1) or 29.2% (control 2) of observed growth defects from single control comparisons were excluded using the average LGR (Fig. 3B, C). Hence, ScreenGarden automatically defines a set of high confidence growth defects for screens based upon two independent controls.

### Quality control using ScreenGarden

**Fig. 4:**
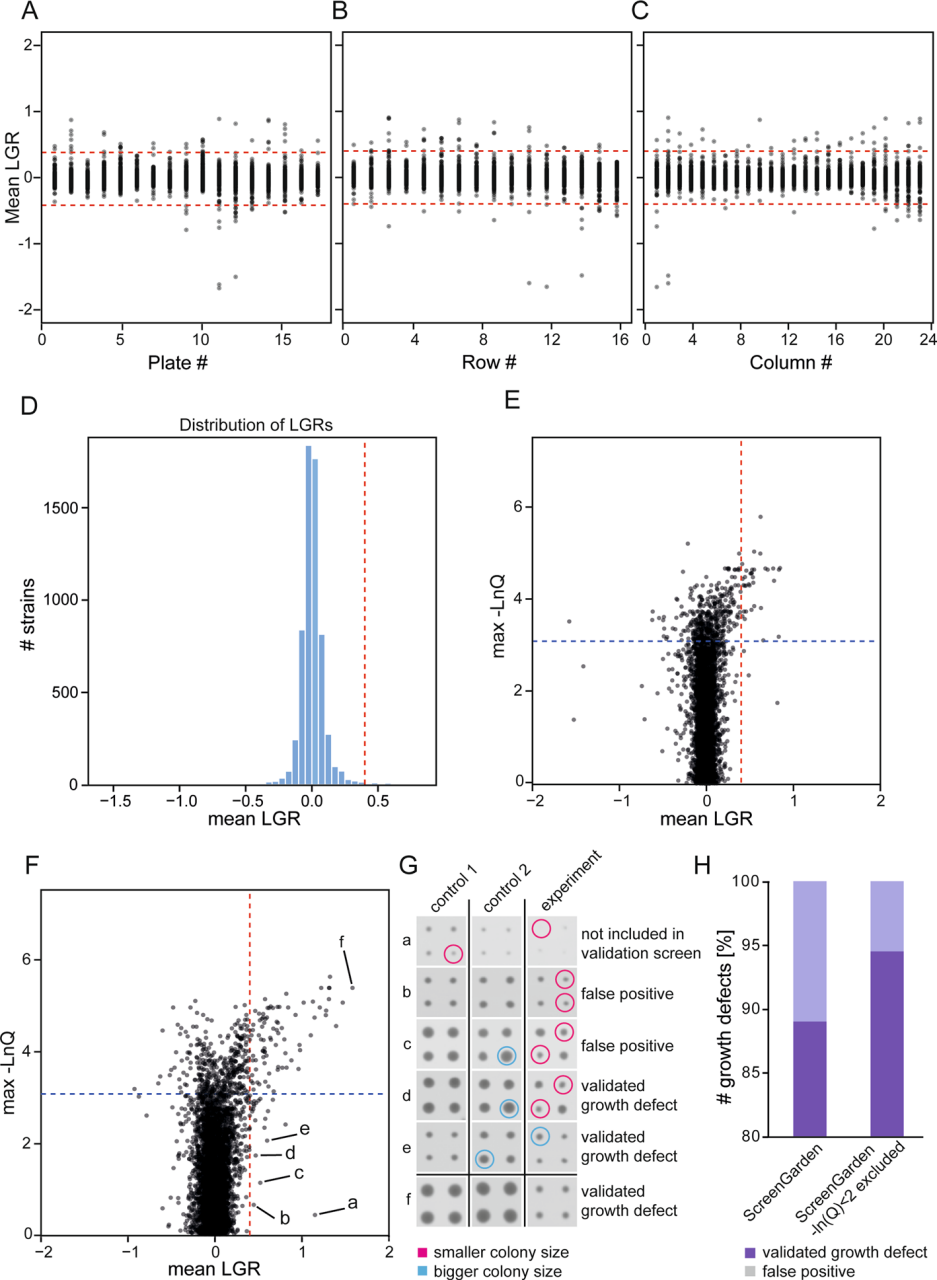
Quality control of plate-based screens using ScreenGarden. Using the ‘Plots’ tab, users can upload.csv files downloaded from ‘ClaculateLGRs’ or ‘Combine2Controls’ and plot any column against each other. For quality control, mean LGRs were plotted against plate (**A**), row (**B**) and column (**C**) number. **D** Histogram showing the distribution of data. The majority of LGRs are distributed close to zero. The red dashed line highlights a LGR of 0.04. **E** Negative max lnQ values were plotted against mean LGRs to identify replicate inconsitencies in the Dad2 Synthetic Physical Interactions dataset. The red dashed line highlights an LGR of 0.04, the black dashed line indicates a max lnQ of 2.99 (q = 0.05). **F** Negative max lnQ values were plotted against mean LGRs to identify replicate inconsitencies in the Nop10 Synthetic Physical Interactions dataset. The data points labelled a to f, most with low max − lnQ values, are analysed in the next panel. **G** Selected growth defects (a to e from panel **F**) with a low max − lnQ value show inconsitencies in colony sizes on plate and 2 of them were identified as false-positive growth defects according to Berry and colleagues [12]. **H** Exclusion based on low max − lnQ values reduced the number of false-positive growth defects

ScreenGarden can be used to plot results directly without laborious reimporting and reformatting in a different application, such as R or Excel. Using the ‘Plots’ tab, either the ‘mean file’, if experiments are compared to one control, or the ‘merge file’, if the screen was performed using two controls, can be uploaded and any two columns can be plotted against each other. Plotting is useful for quality control of screen data, and for example, it allows users to assess the data plate by plate to identify whether any plates produced anomalous LGR values (Fig. 4A). Since the data can be plotted by Row or Column, the data can be scrutinised to ask whether the smoothing algorithm efficiently reduced spatial effects, i.e. whether or not specific rows or columns have higher or lower LGR values (Fig. 4B, C). In the ‘Plots’ function the distribution of LGRs is automatically visualised in a histogram with an adjustable number of bins (Fig. 4D). Plotting mean LGRs against the negative natural logarithm of p- or q-vales respectively allows for rapid assessment of reproducibility, as high p-/q-values account for a large difference in replicate colony sizes. Hence, strains that have inconsistent replicates in the data can be easily identified and if necessary, excluded from further analysis. Only three Synthetic Physical Interactions of the Dad2 dataset were above the q-value threshold of 0.05. We used ScreenGarden to analyse Synthetic Physical Interactions with the nucleolar protein Nop10, a second dataset from Berry and colleagues [12] (Additional file 6). Nop10 association caused a higher number of growth defects compared to the Dad2 dataset, and we found 19 growth defects with a q-value above the threshold (Fig. 4F). Notably, strains with a low value for − lnQ vary in replicate colony sizes (Fig. 4G). We compared these growth defects to the validation screens performed by Berry and colleagues, who identified 7 of these 19 interactions as false positive growth defects (Fig. 4G, H). Exclusion of growth defects based on high q-values decreased the false-discovery rate for the Nop10 screen by approximately 5% (Fig. 4H).

### Comparing ScreenGarden and *ScreenMill*

**Fig. 5:**
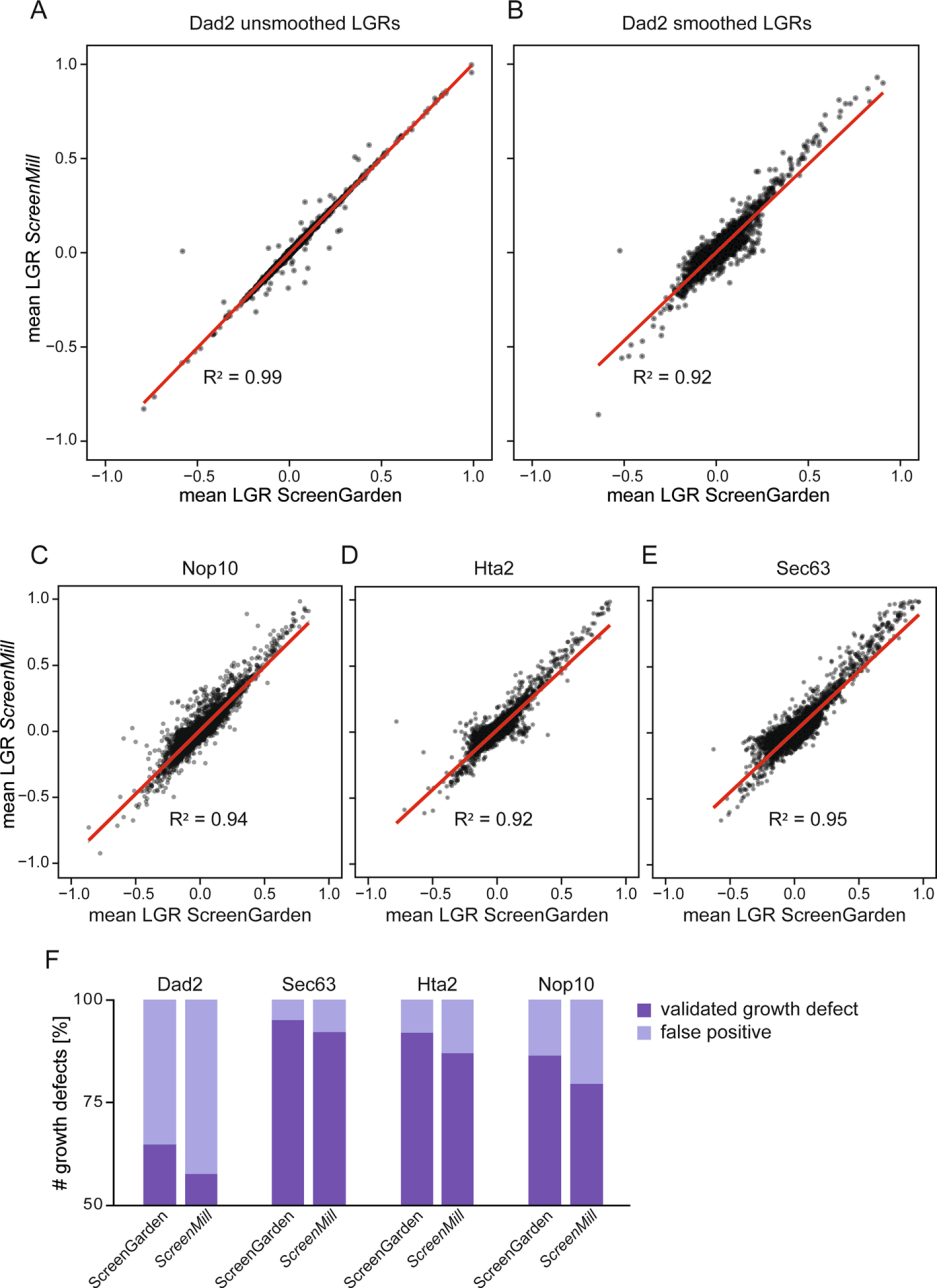
Comparison of ScreenGarden and *ScreenMill*. **A** Unsmoothed data of the Dad2 Synthetic Physical Interactions screen analysed with ScreenGarden and *ScreenMill* are compared. Regression (red line) and Pearson correlation were calculated using the stats 3.6.2. package in RStudio. **B** Smoothed data of the Dad2 Synthetic Physical Interactions screen analysed with ScreenGarden and *ScreenMill* are compared. *ScreenMill* does not automatically smooth data, thus smoothing was performed using PERL based on mean LGRs [5]. Smoothed data of the **C** Nop10, **D** Hta2 and **E** Sec63 Synthetic Physical Interactions screens analysed with ScreenGarden and *ScreenMill* are compared. **F** Both ScreenGarden and *ScreenMill* analysis accounted for similar ratios of true growth defects and false-positives when compared to the validation screen data from Berry and colleagues [12]

Next, we compared the output of ScreenGarden analysis to a previously developed tool for statistical data analysis, the *DR Engine* of the *ScreenMill* software suite (6) (Additional file 7). The *DR Engine* calculates plate median normalised LGRs but does not automatically apply a smoothing algorithm, thus we first compared unsmoothed LGRs from both *ScreenMill* and ScreenGarden (Fig. 5A). As expected, the datasets are highly correlated (R^2^ > 0.99), but, notably, not identical. This observed variance might be due to *ScreenMill’s* automatic exclusion of control-dead colonies for one of the two controls. Control-dead colonies are not excluded in ScreenGarden, but plate normalised colony sizes are reported in the dataset. Since data exclusion is subjective, we allow the user to manually exclude data if the normalised control colony size is below a certain threshold (e.g., 30% of the plate median). A second explanation for the slight variation in data values of ScreenGarden compared to *ScreenMill* is the way LGRs are calculated. The LGRs are used as a measure of growth defect because if, as commonly assumed, the colony sizes are distributed according to a lognormal distribution then the LGRs will be distributed normally. *ScreenMill* calculates the LGR as ln(average control colony size/average experimental colony size) whereas ScreenGarden calculates the LGR for each colony compared to the equivalent position on the control plate before averaging across replicates of the same genotype. This latter approach of applying the logarithm before averaging is more accurate as an approximator of the mean LGR than applying the logarithm to the averaged values, since growth ratios are distributed according to a lognormal distribution and hence LGRs are distributed normally. This effect is generally small but can be significant when the variance between colony sizes is large. Next, we wanted to analyse the effect of automatic data smoothing. Using ScreenGarden, LGRs are smoothed before averaging and independently for each control, whereas *ScreenMill’s* DR engine did not include a smoothing algorithm, hence the data could only be smoothed manually after calculating mean LGRs of two controls. In order to demonstrate the advantage of incorporated data smoothing, we applied our smoothing algorithm to the unsmoothed *ScreenMill* output data and compared this to smoothed ScreenGarden data using four independent datasets (Fig. 5B–E). The smoothed data correlated well for each screen (R^2^ = 0.92–0.95), however, the variance was greater compared to unsmoothed data. Considering that the unsmoothed data correlated almost perfectly, this variance is likely based on the timepoint of smoothing. Last, we analysed the reproducibility of growth defects identified using ScreenGarden and *ScreenMill*. We compared mean LGRs ≥ 0.4 to the results of validation screens performed by Berry and colleagues to distinguish between reproducible growth defects and false-positives (Fig. 5F). Both ScreenGarden and *ScreenMill* performed well in identifying growth defects in the majority of screens, hence we conclude that ScreenGarden analysis can be used to successfully identify reproducible growth defects at least as effectively as *ScreenMill* analysis.

### Defining cut-offs for growth inhibition

**Fig. 6:**
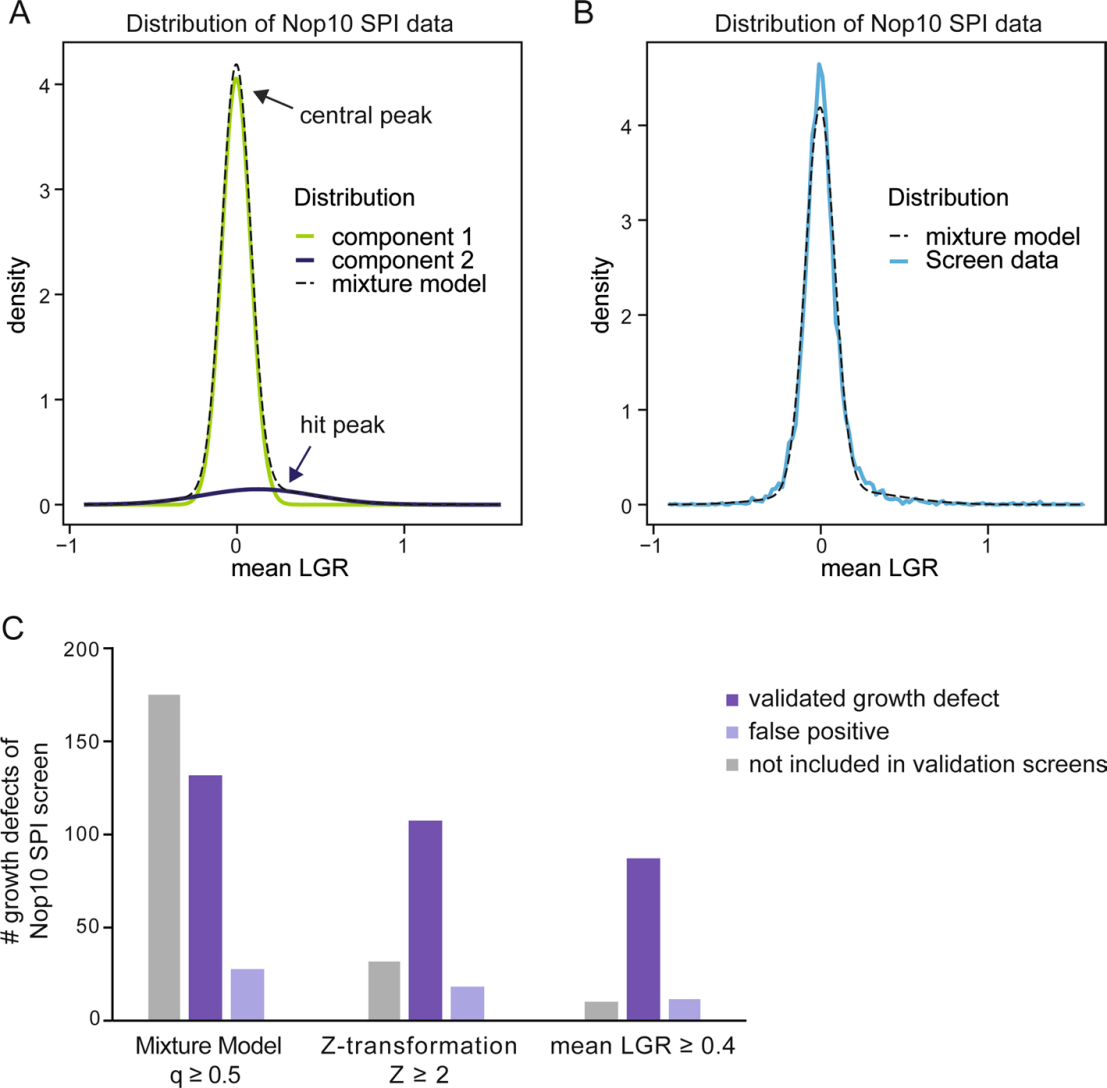
Cut-offs can be defined based on the distribution of screen data. Screens which result in a high number of growth defects are more accurately described using a bimodal mixture model and are characterised by a ‘central peak’ and a second ‘hit peak’. Bimodal mixture models can be fitted automatically to screens with many growth defects using ScreenGarden and produce a component plot (**A**) and a fit plot (**B**) as well as q-values for each LGR. If q ≥ 0.5, the data is predicted to follow the distribution of component 2 and thus LGRs account for predicted growth defects. **C** Mixture model, Z-transformation and empirically defined LGR cut-offs were compared for the Nop10 Synthetic Physical Interactions dataset. Cut-off definition using a bimodal mixture model predicted more than twice the number of growth defects compared to Z-transformation or LGR-based thresholds

Defining the right LGR value or threshold to identify a growth effect varies from screen to screen and is often subjective. However, the threshold choice is important to prevent high rates of false-positives whilst at the same time allowing sensitive identification of growth defects. In this study, we used an empirical cut-off value of LGR = 0.4 to determine growth defects, as previously defined for Synthetic Physical Interaction screens [18, 19]. At LGR = 0.4, a growth defect is moderate but visible compared to control plates. However, ScreenGarden also offers mathematical approaches to define cut-off thresholds, based on the data distribution. ScreenGarden automatically calculates Z-scores, as Z-transformation fits a normal distribution to a dataset and uses the mean and variance of the data to define Z-scores for each data point. The region (− 1.96, 1.96) in Z-space represents 95% of the data in a normal distribution, hence a Z-score above ~ 2 accounts for the strongest 2.5% growth effects within the data. Z-scores have an advantage of allowing datasets to be compared even when they produce quantitatively very different growth effects. However, there are several problems with using Z-scores. First, growth data are typically not normally distributed and second when a normal distribution is applied to a large dataset, there will always be ~ 2.5% of the data with a Z-score > 2 regardless of whether any growth defects were present. Screens that result in some growth defects are likely to display a multimodal or fat-tailed distribution, which is characterised by a longer tail in the positive region of the distribution curve (Fig. 4D). In a previous study, Howell and colleagues have shown that proteome-wide screens such as Synthetic Physical Interactions screens with a high number of growth defects can be described using bimodal normal mixture models [20–22]. Based on the mixture model, the data distribution is composed of two separate components 1 and 2 with distinct peaks (Fig. 6A). Component 1 describes the central peak and contains unaffected strains with LGRs ~ 0, whereas component 2 or the ‘hit peak’ accounts for growth defects with higher LGRs. We have incorporated this script into ScreenGarden in the ‘Mixture Model’ tab, which enables the user to upload their previously calculated ‘mean file’ or ‘merge file’. The bimodal normal mixture model then calculates an FDR-adjusted q-value, with q(x) defined as the probability of inclusion in component 2, given a measured LGR of x. Hence, a q(x) = 0.5 is defined as cut-off point as LGRs are equally likely to be in component 1 or 2. We applied the mixture model fitting to Synthetic Physical Interactions analysis of Nop10, as this screen resulted in a high number of growth defects (Fig. 6A, B) (Additional file 8). We found that q ≥ 0.5 accounted for Synthetic Physical Interactions with an LGR of approximately 0.22 or higher, with a Z-score of as low as 1.3. This led to the identification of more than double the number of growth defects compared to Z-score or LGR-based cut-off definition, however, most of these additional growth defects were not included in validation screens by Berry and colleagues and thus it remains unclear if they are true growth defects or false positives (Fig. 6C). Our findings suggest that using a more conservative cut-off definition, like an empirical value for LGRs when growth is visibly affected, is useful for screens without additional validation to reduce false positives. In contrast, using the bimodal mixture model and subsequent validation screening can extensively increase the number of growth defects identified in screens.

## Discussion

In this study, we introduce ScreenGarden, a shinyR application for rapid statistical analysis pf plate-based high-throughput screens. We have shown that ScreenGarden analysis can be used to report reproducible high-confidence growth defects which are corrected for any potential environmental factors or errors in pinning. We have compared our software to a previously available tool for plate-based screen analysis, *ScreenMill* and have shown that ScreenGarden analysis can identify reproducible growth defects at least as well as *ScreenMill*.

## Conclusion

ScreenGarden is a useful tool for easy, quick and robust analysis of plate-based high throughput assays and facilitates screen analysis that use two independent controls. Data can be plotted immediately without exporting output files into a second application for data visualisation, and ScreenGarden analysis does not require prior experience with handling of large-scale data. ScreenGarden is an open-source shinyR application. All code is written using RStudio and available for download from the ScreenGarden homepage or GitHub. Thus, ScreenGarden can be run not only as a web application but also locally using the open source RStudio software, which runs on Windows, Mac and Linux platforms. This renders the possibility to adapt the code for screen-specific needs and easy customisation of the code. Although here we have only used ScreenGarden for the analysis of SPI data, the software can be used for other analyses that compares plate-based data from experiments and controls. All ScreenGarden tools can be run independently, since the files are directly uploaded for each specific step. The normalisation and smoothing algorithms prevent biases due to plate differences or spatial anomalies, making ScreenGarden a robust tool for data analysis. ScreenGarden can perform analysis within seconds and provides data visualisation. Plots can be downloaded as PDF files for further preparation or directly incorporated into presentations or reports. Conclusively, ScreenGarden provides an easy to use software tool for plate-based microbial screen analysis.

### Yeast strains and methods

The data for validation (Figs. 4, 5, 6) were previously published [12]. The methods and strains are described in this publication, but briefly, the universal donor strain (UDS) is an *ADE2*^+^ *RAD5*^+^ derivative of W303 (*can1-100 his3-11,15 leu2-3,112 ura3-1*) unless otherwise indicated [23]. GFP strains are all based upon BY4741 (*his3∆1 leu2∆0 met15∆0 ura3∆0*) [24]. Yeast were grown in standard growth medium including 2% (weight/volume) of the indicated carbon source [25]. Plasmids for SPI screens (encoding GBP alone, protein alone or protein-GBP) were created using the gap-repair cloning technique, which combines a linearized plasmid with PCR products using in vivo recombination. All PCR products were generated using primers from Sigma Life Science and Q5 polymerase (New England Biolabs, USA). All plasmid constructs were validated using Sanger sequencing (Beckman Coulter Genomics, UK). The SPI screens utilised Selective Ploidy Ablation (SPA) methodology, a mating-based approach for yeast transformation [26]. In the first step, SPI plasmid constructs were separately transferred into the UDS by transformation. Each of these strains is then mated individually to the collection of GFP strains. These diploid cells are converted back to haploids by both destabilizing and counter-selecting against all the UDS chromosomes. The resulting haploid colonies, containing the specific plasmids, are then assessed for growth by measuring colony size. All mating and copying of the yeast colonies were performed using a RoToR pinning robot (Singer Instruments, UK).

### Availability and requirements

Project name: ScreenGarden.

Project home page: https://screengarden.shinyapps.io/screengardenapp/.

R scripts available to download from the homepage or via GitHub: https://github.com/CinziaK/ScreenGarden.

Operating system: Platform independent or Mac, Windows, Linux for the standalone application using RStudio.

Programming language: R.

License: GNU GPL.

Any restrictions to use by non-academics: none.

## Supplementary Information

**Additional file 1.** Detailed instructions on how to run ScreenGarden data analysis using the web application or locally in RStudio.

**Additional file 2.** Example of a ‘Log file’ produced by *ScreenMill’s* CM-Engine. Colony size and circularity are reported as a list ordered by plate position and number of the plate.

**Additional file 3.** Example of an alternative colony size input file recognised by ScreenGarden, containing information about the query and control names (Label), plate position (Plate, Row, Column) and colony sizes (colonysize).

**Additional file 4.** Example of a ‘key file’ that annotates the genotype of strains (ID) to their specific position on the plates.

**Additional file 5.** Genome-wide Synthetic Physical Interactions screens with the outer kinetochore protein Dad2 were analysed using the ‘CalculateLGRs’ and ‘Combine2Controls’ tools of ScreenGarden. Column headers are described in detail in Additional file 1.

**Additional file 6.** Genome-wide Synthetic Physical Interactions screens with the outer kinetochore protein Nop10 were analysed using the ‘CalculateLGRs’ and ‘Combine2Controls’ tools of ScreenGarden. Column headers are described in detail in Additional file 1.

**Additional file 7.** Comparison of SPI data analysis using ScreenGarden and *ScreenMill.* Since *ScreenMill* analysis does not include automatic smoothing of plate effects, *ScreenMill* data was smoothed manually using ScreenGarden’s smoothing algorithm. SPI data were compared for screens with Dad2, Nop10, Hta2 and Sec63.

**Additional file 8.** The data reports the results of mixture model fitting for SPI data with Nop10 using ScreenGarden’s ‘Mixture Model’ tool. Mixture model fitting produces q-values for each mean LGR. A q-value > 0.5 indicates that SPIs are likely to be distributed in component 2, representing growth defects.

## Data Availability

All data and materials are included in the supplementary files or can be accessed via the ScreenGarden web page: https://screengarden.shinyapps.io/screengardenapp/.

## Abbreviations

LGR: Log growth ratio
GFP: Green fluorescent protein
GBP: GFP-binding protein
FDR: False discovery rate
ID: Identifier

## References

[1] Boone C, Bussey H, Andrews BJ (2007). Exploring genetic interactions and networks with yeast. Nat Rev Genet.

[2] Gavin AC, Aloy P, Grandi P, Krause R, Boesche M, Marzioch M (2006). Proteome survey reveals modularity of the yeast cell machinery. Nature.

[3] Krogan NJ, Cagney G, Yu H, Zhong G, Guo X, Ignatchenko A (2006). Global landscape of protein complexes in the yeast *Saccharomyces cerevisiae*. Nature.

[4] Ito T, Chiba T, Ozawa R, Yoshida M, Hattori M, Sakaki Y (2001). A comprehensive two-hybrid analysis to explore the yeast protein interactome. Proc Natl Acad Sci USA.

[5] Ólafsson G, Thorpe PH (2015). Synthetic physical interactions map kinetochore regulators and regions sensitive to constitutive Cdc14 localization. Proc Natl Acad Sci USA.

[6] Dittmar JC, Reid RJ, Rothstein R (2010). Open access SOFTWARE ScreenMill: a freely available software suite for growth measurement, analysis and visualization of high-throughput screen data. BMC Bioinform.

[7] Lamprecht MR, Sabatini DM, Carpenter AE (2007). Cell profiler: free, versatile software for automated biological image analysis. Biotechniques.

[8] Wagih O, Parts L (2014). Gitter: a robust and accurate method for quantification of colony sizes from plate images. G3.

[9] Bischof L, Převorovský M, Rallis C, Jeffares DC, Arzhaeva Y, Bähler J (2016). Spotsizer: high-throughput quantitative analysis of microbial growth. Biotechniques.

[10] RStudio I. Easy web applications in R. 2013.

[11] RStudio Team. RStudio: integrated development for R. RStudio, Inc; 2015. http://www.rstudio.com/.

[12] Berry LK, Ólafsson G, Ledesma-Fernández E, Thorpe PH (2016). Synthetic protein interactions reveal a functional map of the cell. Elife.

[13] Perlstein EO, Deeds EJ, Ashenberg O, Shakhnovich EI, Schreiber SL (2007). Quantifying fitness distributions and phenotypic relationships in recombinant yeast populations. Proc Natl Acad Sci.

[14] Collins SR, Schuldiner M, Krogan NJ, Weissman JS (2006). A strategy for extracting and analyzing large-scale quantitative epistatic interaction data. Genome Biol.

[15] Baryshnikova A, Costanzo M, Kim Y, Ding H, Koh J, Toufighi K (2010). Quantitative analysis of fitness and genetic interactions in yeast on a genome scale. Nat Methods.

[16] Benjamini Y, Hochberg Y, Benjamini Y, Hochberg Y (1995). Controlling the false discovery rate: a practical and powerful approach to multiple testing. J R Stat Soc B.

[17] Fisher RA (1925). Statistical methods for research workers.

[18] Ólafsson G, Thorpe PH (2020). Polo kinase recruitment via the constitutive centromere-associated network at the kinetochore elevates centromeric RNA. PLoS Genet.

[19] Ólafsson G, Thorpe PH (2016). Synthetic physical interactions map kinetochore-checkpoint activation regions. G3 Genes Genomes Genet.

[20] Howell RSM, Csikász-Nagy A, Thorpe PH (2019). Synthetic physical interactions with the yeast centrosome. G3 Genes Genomes Genet.

[21] Fraley C, Raftery AE (2002). Model-based clustering, discriminant analysis, and density estimation. J Am Stat Assoc.

[22] Scrucca L, Fop M, Murphy TB, Raftery AE (2016). Mclust 5: clustering, classification and density estimation using Gaussian finite mixture models. R J.

[23] Zou H, Rothstein R (1997). Holliday junctions accumulate in replication mutants via a RecA homolog-independent mechanism. Cell.

[24] Huh WK, Falvo JV, Gerke LC, Carroll AS, Howson RW, Weissman JS (2003). Global analysis of protein localization in budding yeast. Nature.

[25] Sherman F (2002). Getting started with yeast. Methods Enzymol.

[26] Reid RJD, González-Barrera S, Sunjevaric I, Alvaro D, Ciccone S, Wagner M (2011). Selective ploidy ablation, a high-throughput plasmid transfer protocol, identifies new genes affecting topoisomerase I-induced DNA damage. Genome Res.

