# Supplementary material for: ScreenGarden: a shinyR application for fast and easy analysis of plate-based high-throughput screens
Source: BMC Bioinformatics. 2022 Feb 5;23:60. doi: 10.1186/s12859-022-04586-1 (PMC8818250; doi:10.1186/s12859-022-04586-1)
Supplement: Supplementary file 1 — Additional file 1. Detailed instructions on how to run ScreenGarden data analysis using the web application or locally in RStudio. [file 12859_2022_4586_MOESM1_ESM.pdf]

User Guide for data analysis using ScreenGarden

This is the user guide for data analysis using the ScreenGarden software written by Cinzia Klemm, Rowan Howell and Peter Thorpe in 2020.

Contents

1. General 1
2. Deploying ScreenGarden offline in RStudio 2
   2.1. Packages 2
   2.2. ScreenGarden Code 2
   2.3. Deploying ScreenGarden 2
3. Deploying ScreenGarden via the Shinyapps website 2
4. Using ScreenGarden 3
   4.1. ScreenGarden Homepage 3
   4.2. Calculate LGRs 3
      4.2.1. Rationale 3
      4.2.2. How to use 'Calculate LGRs' 3
   4.3. Combine2Controls 5
      4.3.1. Rationale 5
      4.3.2. How to use 'Combine2Controls' 5
   4.4. Plotting data using ScreenGarden 5
      4.4.1. Rationale 5
      4.4.2. How to use 'Plots' 5
   4.5. Mixture Model Fitting 6
      4.5.1. Rationale 6
      4.5.2. How to use 'Mixtrue Model' 7
5. Input file requirements 8
   5.1. Colony size file 8
      5.1.1. CM-engine output 8
      5.1.2. Other colony size input files 8
   5.2. Key files 9
6. Output files 9
   6.1. 'Calculate LGRs' output 9
   6.2. 'Combine2Controls' output 10
   6.3. 'Mixture Model' output 10
7. Additional Information 11

1. General

The ScreenGarden data analysis software can be deployed directly from the ScreenGarden shinyapps website or locally using RStudio by downloading the R code from the website or GitHub (see 2.2.).
If you would like to download ScreenGarden and run it locally in RStudio please follow the instructions

in **section 2** to download and deploy ScreenGarden.
If you would like to perform ScreenGarden analysis from the ScreenGarden shinyapps website, please skip to **section 3**.

2. Deploying ScreenGarden offline in RStudio

ScreenGarden analysis has been successfully tested in RStudio, an easy interface for the programming language R. Running ScreenGarden directly in RStudio enables offline data analysis. The following steps will explain how to deploy ScreenGarden in RStudio. Additionally, a video tutorial of how to set up ScreenGarden in RStudio is provided as supplementary information.

2.1. Packages

ScreenGarden requires specific R packages to perform data analysis. It is necessary to install the required packages for ScreenGarden analysis. For example, this can be done by entering the following code into the console of RStudio:

```
install.packages(c("tidyverse","lubridate","rlang","ggplot2","Cairo","gghighlight","shiny","shinythemes","png","mclust"))
```

Alternatively, the user can manually select the following packages with the path Tools >> Install packages:

- tidyverse
- lubridate
- rlang
- ggplot2
- Cairo
- gghighlight
- shiny
- shinythemes
- png
- mclust

2.2. ScreenGarden Code

ScreenGarden scripts can be downloaded from the ScreenGarden website (https://screengarden.shinyapps.io/screengardenapp/) using the 'Download R scripts' button or from GitHub (https://github.com/CinziaK/ScreenGarden).

2.3. Deploying ScreenGarden

Open the server.R and ui.R code in R studio (File >> Open File…).
Press "Run app" (Figure1). A second window will appear which shows the ScreenGarden user interface (the same as for the web version).

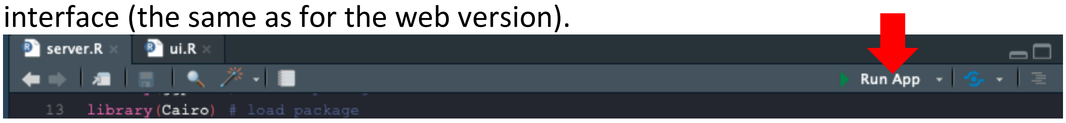

Figure 1: The cropped image shows a screenshot of the top of the RStudio interface after opening the server.R and ui.R scripts. The red arrow indicates how to run the app.

3. Deploying ScreenGarden via the Shinyapps website

ScreenGarden analysis via the shinyapps website (https://screengarden.shinyapps.io/screengardenapp/) does not require any previous installations and

was tested on several commonly used web browser (Chrome, Firefox and Safari).
Please note that sessions will time out after 5 minutes.

4. Using ScreenGarden

4.1. ScreenGarden Homepage

Both, running ScreenGarden in RStudio or via the shinyapps website, produces the same user interface for screen analysis using ScreenGarden (Figure 2). The home page contains download buttons for the Instructions (this document), the ScreenGarden R scripts to deploy ScreenGarden from RStudio, and example files to use with the ScreenGarden software. A video tutorial demonstrating how to use ScreenGarden is provided as supplementary data.

Figure 2: Homepage
The ScreenGarden homepage contains download buttons for the Instructions, R scripts and example files, indicated by the red arrows.

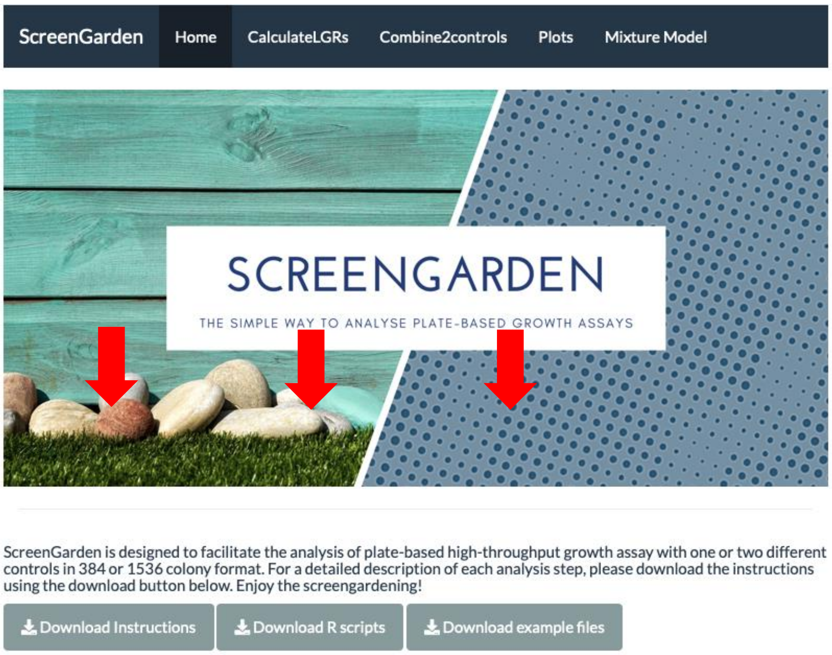

4.2. Calculate LGRs

4.2.1. Rationale

The 'Calculate LGRs' tool is designed to calculate Log Growth Ratios (LGRs); the natural logarithm of the size of the control colonies divided by the experimental colonies (Figure 3). ScreenGarden also calculates Z-scores, *p*- and *q-values* for each mean LGR. Z-scores describe the position of an LGR value in a normal distribution and can be used as a cut-off for growth defects if the data is normally distributed. A T-test is used to calculate *p-values* comparing individual colony replicates with each other, indicating the reproducibility of growth effects. The user can also choose to evaluate LGR reproducibility based on FDR-corrected *q-values*, which are calculated from p-values using the Benjamini and Hochberg method (Benjamini & Hochberg, 1995).

4.2.2. How to use 'Calculate LGRs'

The user can easily import input colony size and key files (see Section 5). The control and query name depend on the name in their input files (see Section 5). Colony size input files can either be derived from *ScreenMill's* CM-engine or imported in the specific format as described in Section 5. Replicate numbers can be set to 1, 4 or 16 (which refers to how many times each strain is replicated on the plate), and array size can be set to 384 or 1536 (which refers to the number of colonies on the plates). Note that the default settings are for colonyAreas.txt files generated by CM-engine, with 4 replicates and 1536

colonies per plate. The user can choose between plate normalisation based on the plate median (default) or the mean of a positive control (defined as 'Control' in the key file, see Section 5). Plates can be optionally smoothed based on row and column median growth rates (smoothing is selected by default). Plate median normalisation and smoothing should only be selected if most query colonies are **not affected** compared to controls colonies in the screen.

Two output files can be produced by the Calculate LGRs tool, a 'mean file' (shown when 'Display' is set to 'Means') and a 'replicates file' (shown when 'Display' is set to 'Replicates'). The 'mean file' produces a list of LGRs and *p/q-values* for the average of the replicates, whereas all information for each replicate is listed in the 'replicates file' (see Section 6).

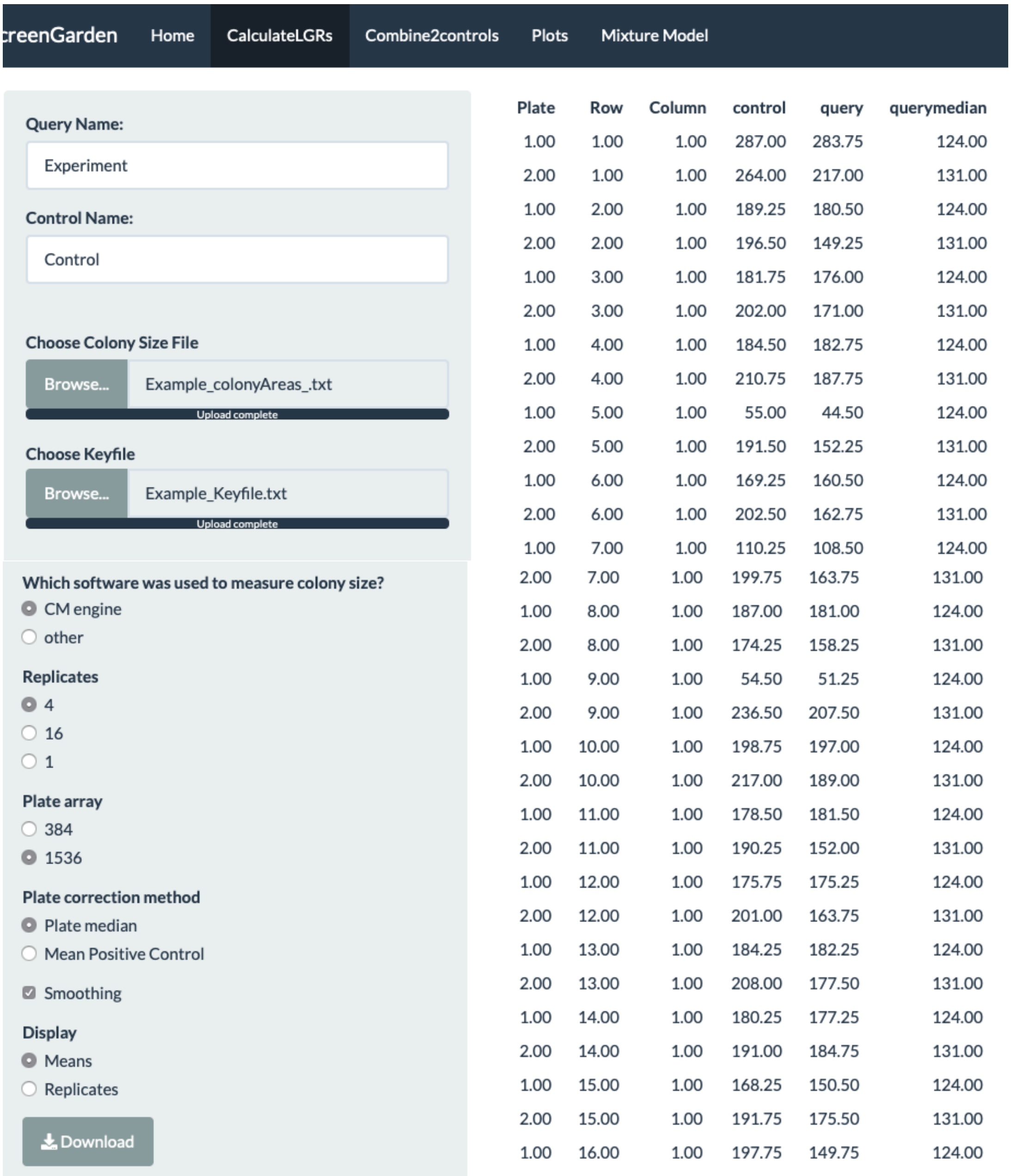

Plate	Row	Column	control	query	querymedian
1.00	1.00	1.00	287.00	283.75	124.00
2.00	1.00	1.00	264.00	217.00	131.00
1.00	2.00	1.00	189.25	180.50	124.00
2.00	2.00	1.00	196.50	149.25	131.00
1.00	3.00	1.00	181.75	176.00	124.00
2.00	3.00	1.00	202.00	171.00	131.00
1.00	4.00	1.00	184.50	182.75	124.00
2.00	4.00	1.00	210.75	187.75	131.00
1.00	5.00	1.00	55.00	44.50	124.00
2.00	5.00	1.00	191.50	152.25	131.00
1.00	6.00	1.00	169.25	160.50	124.00
2.00	6.00	1.00	202.50	162.75	131.00
1.00	7.00	1.00	110.25	108.50	124.00
2.00	7.00	1.00	199.75	163.75	131.00
1.00	8.00	1.00	187.00	181.00	124.00
2.00	8.00	1.00	174.25	158.25	131.00
1.00	9.00	1.00	54.50	51.25	124.00
2.00	9.00	1.00	236.50	207.50	131.00
1.00	10.00	1.00	198.75	197.00	124.00
2.00	10.00	1.00	217.00	189.00	131.00
1.00	11.00	1.00	178.50	181.50	124.00
2.00	11.00	1.00	190.25	152.00	131.00
1.00	12.00	1.00	175.75	175.25	124.00
2.00	12.00	1.00	201.00	163.75	131.00
1.00	13.00	1.00	184.25	182.25	124.00
2.00	13.00	1.00	208.00	177.50	131.00
1.00	14.00	1.00	180.25	177.25	124.00
2.00	14.00	1.00	191.00	184.75	131.00
1.00	15.00	1.00	168.25	150.50	124.00
2.00	15.00	1.00	191.75	175.50	131.00
1.00	16.00	1.00	197.75	149.75	124.00

Figure 3: Calculate LGRs

The 'Calculates LGRs' tab can be used to calculate LGRs relative to a control. Using the example colonyAreas.txt file and example key file the user can run the script by entering the query name 'Experiment' and control name 'Control' to calculate LGRs. Note that the names 'Experiment' and 'Control' are specific to the example dataset. In order to use their own data users will have to enter Query and Control names that match those in their colony size file (see Section 5). The user can choose the format of the colony size input file, the number of replicates, the array size, the plate correction method (with and without smoothing) and which file is displayed and downloaded.

To use the example data provided, default settings can be used.

4.3. Combine2Controls

4.3.1. Rationale

For screens with two controls, two data files produced by the ‘Calculate LGRs’ tool can be combined automatically, calculating the mean LGRs of two independent controls compared to the same experimental colonies (Figure 4). In addition, ‘Combine2Controls’ produces mean Z-scores and max p/q values. Max p-and q-values are defined as the higher p-or q-value calculated for each control comparison.

4.3.2. How to use ‘Combine2Controls’

The user can simply upload two ‘mean files’ produced by the ‘Calculates LGR’ tool to automatically combine screen data with two controls. The resulting ‘merge file’ can be downloaded via the ‘Download’ button.

Figure 4: Combine2Controls
Two ‘mean files’ produced by the Calculates LGR tool can be combined for screen analysis with two controls.

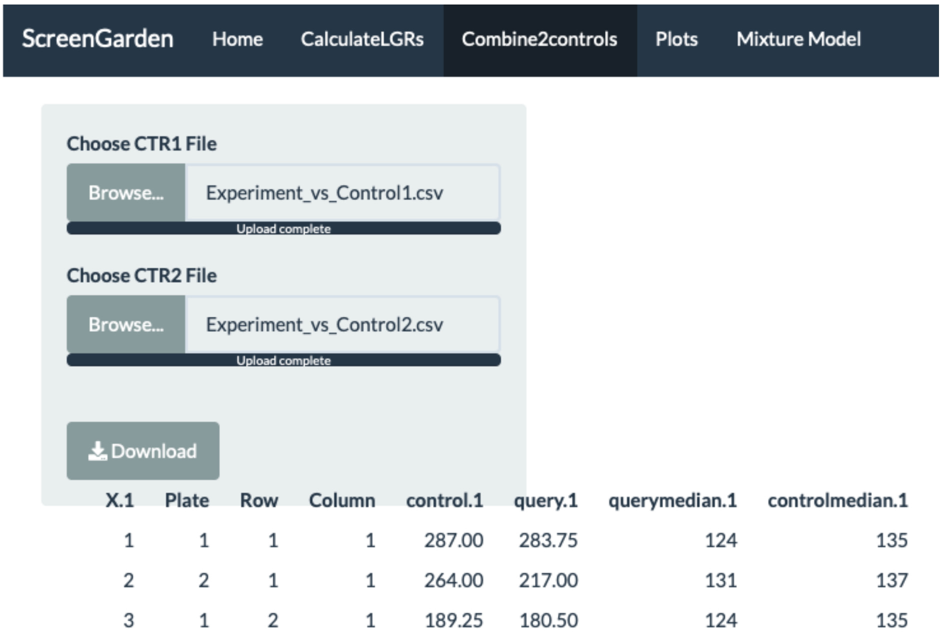

4.4. Plotting data using ScreenGarden

4.4.1. Rationale

Output data from both, the ‘Calculates LGRs’ tool and the ‘Combine2Controls’ tool can be uploaded into the ‘Plots’ tab. This enables plotting of any of the columns to another column of the dataset. For example, mean LGRs can be plotted against the negative logarithm of *q-values*, which produces a volcano plot, indicating the strength of the growth defect (LGR) and its reproducibility (-log(q)) (Figure 5). Plots can be downloaded in .pdf format via the ‘Download’ button. Furthermore, a histogram shows the distribution of the data below the plot (Figure 5).

4.4.2. How to use ‘Plots’

The user can upload the data by using the ‘choose csv file’ button and select a ‘mean file or ‘merge file’. Next, the user has to define the X and Y-variables with the 2 pulldown menues to generate plots. Bin numbers of the histogram can be adjusted with the ‘number of bins’ cursor.

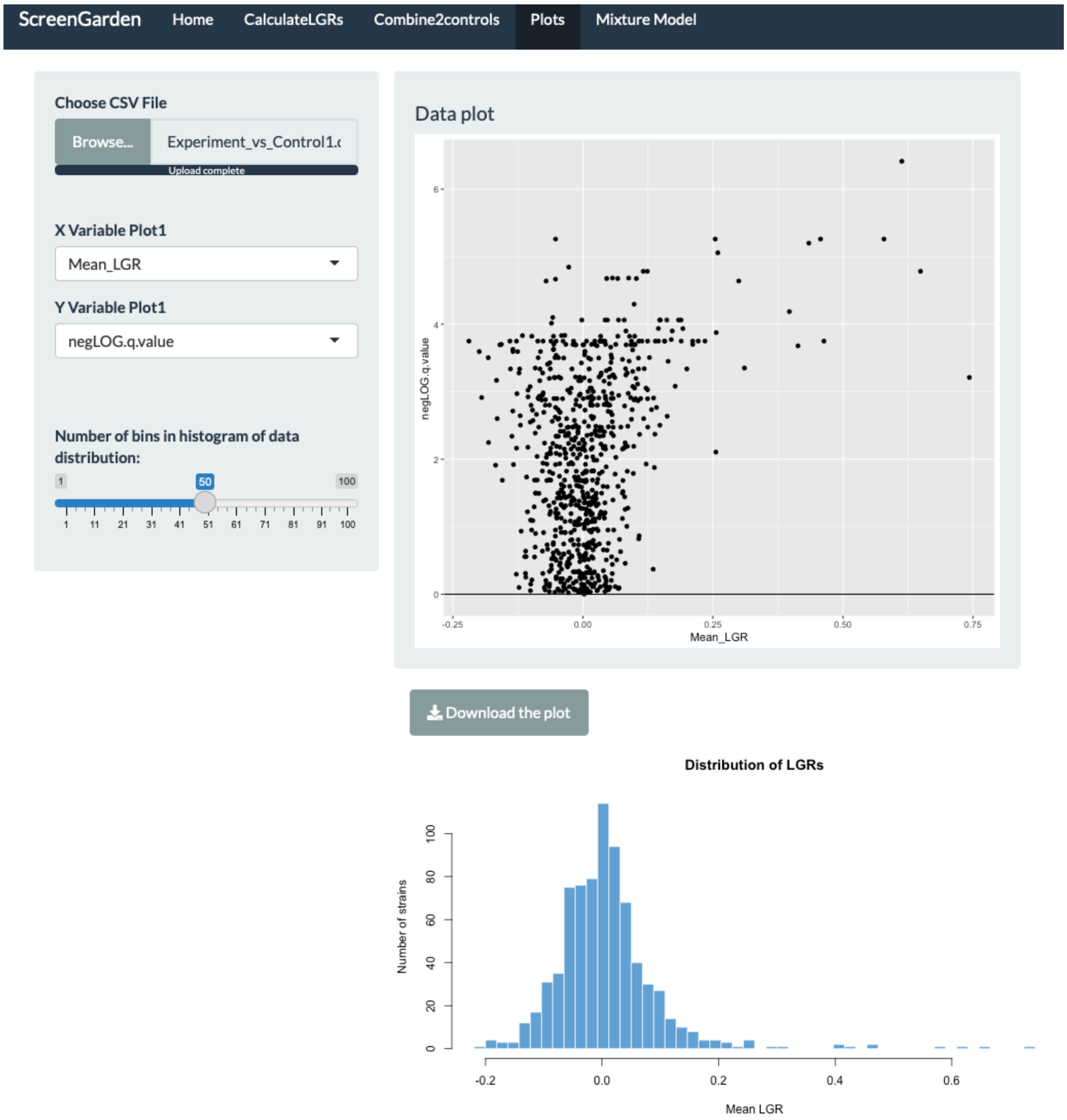

Figure 5: Plotting using the ScreenGarden software.
Output 'mean files' (from 'Calculates LGRs') and 'merge files' (from 'Combine2Controls') can be reimported into the plots tab and plotted by any column. The example shows Mean LGRs plotted against -ln(q) values. The histogram shows the distribution of the data and bin number can be adjusted on the left-hand panel.

4.5. Mixture Model Fitting

4.5.1. Rationale

Growth defect thresholds based on Z-scores are less suitable for large-scale screening data, as the data is often not normally distributed and contains a longer 'positive tail' (Howell et al., 2019). The data distribution is better described by a bimodal model, and mere Z-score evaluation can lead to exclusion of subtle, but true growth defects. We have incorporated the mixture model fitting script into ScreenGarden, to easily define thresholds based on the bimodal mixture model (Figure 6). A bimodal

distribution consists of 2 components, Component 1 and 2. Component 1 describes LGRs of colonies which are not affected in growth compared to control colonies, whereas LGRs distributed in Component 2 are likely to be affected in growth. ScreenGarden can be used to calculate *q-values* (referred to as '*q-vals*'), which define if LGRs are distributed in Component 1 or Component 2. A *q-val* > 0.5 describes a likelihood of an LGR value being in the distribution of affected strains (Component 2). This tool also produces a 'Density' and a 'Component' plot, which visualise the bimodal distribution for the input data.

4.5.2. How to use 'Mixtrue Model'

Mixture model fitting for data files from the 'Calculates LGRs' and 'Combine2Controls' tools can be performed by simply selecting the file. The 'Density' and 'Component plot' will appear automatically. Additionally, a list of *q-vals* is produced for each LGR and can be downloaded via the download button. Note that an Error message will appear when mixture model fitting is unsuccessful. This is the case when the distribution is better described by a normal distribution, e.g. when there are approximately as many positive as negative LGRs. In this instance, a mixture model should not be used.

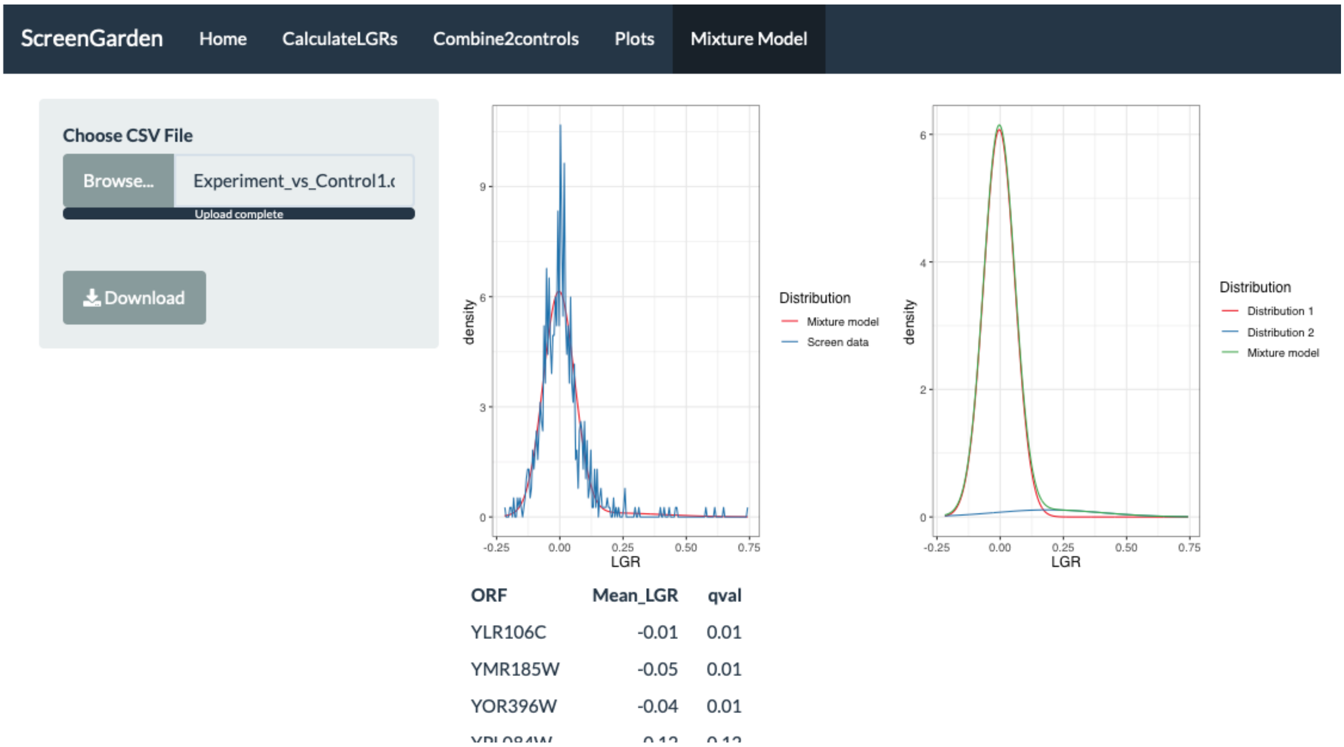

Figure 6: Mixture model fitting using ScreenGarden.
The Mixture model tab can be used to define thresholds for growth defects based on a bimodal mixture model distribution and produces a 'Density' plot (left) and a 'Component' plot (right). Q-vals greater than 0.5 are distributed in Component 2 and describe growth defects.

5. Input file requirements

5.1. Colony size file

ScreenGarden recognises two types of colony size files for statistical data analysis. First, *ScreenMill's* CM-engine output file ('colonyAreas.txt') (Dittmar et al., 2010) and second, a file containing colony size, plate and experiment information (see Section 5.1.2). Example files can be downloaded from the ScreenGarden homepage.

5.1.1. CM-engine output

ScreenMill's CM-engine produces a 'log-file' named 'colonyAreas.txt' listing colony size and circularity (Figure 7). The different plates and experiment or control identifier are included along the list in specific rows (e.g. 1536 array in row 1, row 1538, row 3075…). The 'colonyAreas.txt' file can be directly uploaded into ScreenGarden. 'Query name' and 'Control name' should be carefully noted as they are needed to define which conditions to compare in ScreenGarden (see blue box in Figure 7).

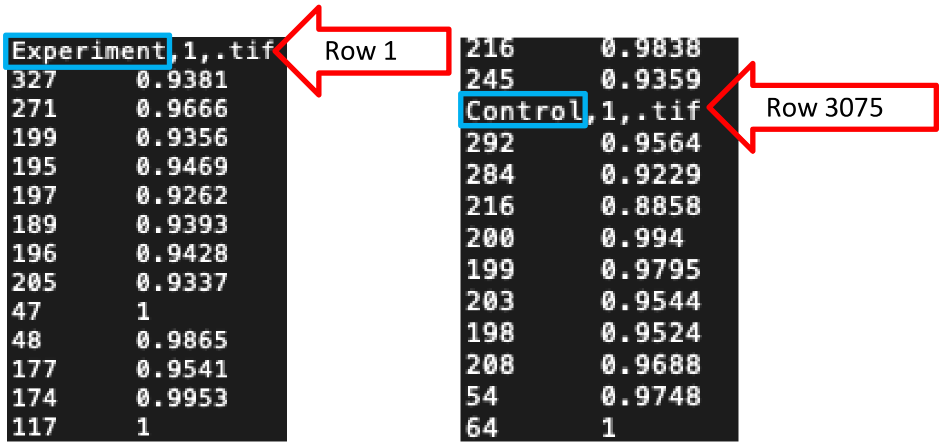

Figure 7. Example of the CM-engine output

The crops show the colonyAreas.txt output file derived from *ScreenMill's* CM-engine. Blue boxes indicat names for query and control in the ScreenGarden 'Calculate LGRs' tab. Red arrows indicate row numbers.

5.1.2. Other colony size input files

If a different file formt is used for ScreenGarden analysis, the box for "other' has to be ticked in the 'Calculate LGRs' tab. The format of this input file has to be as shown in Figure 8. The 'Calculate LGRs' tool is case sensitive. The Column 'Label' should be either the name of the experiment or the name of the control and should be identical for all colonies on a plate.

Label	Plate	colonysize	Row	Column
Experiment	1	72	1	1
Experiment	1	266	2	1
Experiment	1	238	3	1
Experiment	1	219	4	1
Experiment	1	218	5	1
Experiment	1	210	6	1
Experiment	1	222	7	1
Experiment	1	221	8	1
Experiment	1	48	9	1
Experiment	1	55	10	1
Experiment	1	204	11	1
Experiment	1	216	12	1
Experiment	1	172	13	1
Experiment	1	181	14	1
Experiment	1	185	15	1
Experiment	1	207	16	1
Experiment	1	61	17	1
Experiment	1	63	18	1
Experiment	1	247	19	1
Experiment	1	226	20	1

Figure 8: Alternative Input file type.

Alternative to CM-engine output files, custom colony size files can be uploaded into ScreenGarden. These files have to contain a column that defines the 'Label' of the plate (here Experiment or Control (the latter is not shown)), a column for the plate number ('Plate'), a column for the colony size value ('colonysize') a column for the row number ('Row') and a column for the column number ('Column')

5.2. Key files

ScreenGarden analysis requires a key file, which identifies the names of strains and their position on the plates. The definition for the key is defined by the 'ID' column (here using systematic gene names of *Saccharomyces cerevisiae*) each linked to a specific plate, row and column. The first rows of an example key file are shown in Figure 9. The values for 'Plate', 'Row' and 'Column' must be numeric, whereas 'ID' values can be represented by letters. The header names are case sensitive. If 'normalisation based on mean control' colonies is selected, these controls need to be identified as 'Control' in the ID column.

Plate	Row	Column	ID
1	1	1	YLR106C
1	1	2	YLL021W
1	1	3	YLR454W
1	1	4	YDL112W
1	1	5	YJL130C
1	1	6	YCR081W
1	1	7	YKL182W
1	1	8	YPR164W
1	1	9	YNL339C
1	1	10	YIL159W
1	1	11	YBR208C
1	1	12	YAL017W
1	1	13	YNL172W
1	1	14	YCR073C
1	1	15	YOR341W
1	1	16	YDR159W
1	1	17	YGR098C
1	1	18	YMR076C
1	1	19	YOR326W
1	1	20	YJL197W

Figure 9: Example of a key file.
Key files contain information about the plate, row and column position of colonies and their identifier names in the column labelled 'ID'. In this example, the identifiers are systematic gene names from *S. cerevisiae.*

6. Output files

All ScreenGarden output data files are saved as comma-separated values (CSV) files. These can be opened in Excel, R or other software for data handling. Note that ScreenGarden data files contain the values of all intermediate calculation steps for transparency. The following list describes the column headers of the 'mean file' produced by 'Calculate LGRs' (Table 1). These are identical in the 'replicates file', but values of individual replicates are split into separate columns (e.g. mean_LGR.1, mean_LGR.2..).

6.1. 'Calculate LGRs' output

Table 1: Description of 'mean file' headers produced by 'Calculate LGRs'

Column Header	Description
Plate	Plate number
Row	Row number
Column	Column number
control	Colony size of control
query	Colony size of Query
querymedian	Median of Query colony size (if median corrected, otherwise mean of Control)
controlmedian	Median of Control colony size (if median corrected, otherwise mean of Control)

norm_query	Normalised query colony size (query/querymedian)
norm_control	Normalised control colony size (control/controlmedian)
log_norm_query	natural log of the normalised query colony size
log_norm_control	Natural log of the normalised control colony size
mean_usLGR	Mean of unsmoothed log growth ratio (LGR)
mean_LGR	Mean of smoothed log growth ratio (LGR)
sd_usLGR	Standard deviation of unsmoothed log growth ratio (LGR)
sd_LGR	Standard deviation of smoothed log growth ratio (LGR)
p.value	*p-value* of replicates
q.value	FDR corrected *q-value* calculated from p-values (Benjamini and Hochberg method)
negLOG.p.value	Negative natural log of *p-values* (*p-value* = 0.05 is approx. -ln(*p*) = 3)
negLOG.q.value	Negative natural log of *q-values* (*q-value* = 0.05 is approx. -ln(*q*) = 3)
mean_Z_score	Mean Z-scores of LGRs, can be used to evaluate growth for normally distributed data
ID	Identifies colony genotype (e.g. gene name)

6.2. 'Combine2Controls' output

The 'Combine2Controls' script combines two 'mean file' data files. The output 'merge file' contains the information of both input data files (see Table 1) with the suffixes '.1' and '.2' for each control comparison data and adds the following columns listed in Table 2 at the end of the dataset.

Table 2: Description of 'merge file' headers produced by 'Combine2Controls'

Column Header	Description
Mean_LGR	Mean LGRs combined from both control comparisons
Mean_Z	Mean LGRs combined from both control comparisons
max_p	Maximum *p-value* (the higher of the *p-values* from individual control comparisons)
max_q	Maximum *q-value* (the higher of the *q-values* from individual control comparisons)
max_neglLOG.p.value	Maximum negative natural log of *p-values* (*p-value* = 0.05 is approx. -ln(*p*) = 3)
max_negLOG.q.value	Maximum negative natural log of *q-values* (*q-value* = 0.05 is approx. -ln(*q*) = 3)

6.3. 'Mixture Model' output

The output data file produced from mixture model fitting via the 'Mixture Model' tab is reduced to the ID identifier, mean LGRs and *q-vals*, defining the component in which the data is likely to be distributed (Table 3).

Table 3: Description of 'mixture model file' headers produced by 'Mixture Model'

Column Header	Description
ID	Identifies colony genotype
Mean_LGR	Mean LGRs
qval	*q-val* defining if an LGR is likely to be distributed in Component 1 (*q-val* < 0.5) or Component 2 (*q-val* ≥ 0.5) of a bimodal distribution.

7. Additional Information

Thank you for choosing ScreenGarden for data analysis of high-throughput plate-based screens. For problems with the website or R-code or general enquiries please contact Cinzia Klemm via GitHub https://github.com/CinziaK or contact Peter Thorpe via p.thorpe@qmul.ac.uk.

References

Benjamini, Y., & Hochberg, Y. (1995). Benjamini Y, Hochberg Y. Controlling the false discovery rate: a practical and powerful approach to multiple testing. *Journal of the Royal Statistical Society B*, *57*(1), 289–300. https://doi.org/10.2307/2346101

Dittmar, J. C., Reid, R. J., & Rothstein, R. (2010). *Open Access SOFTWARE ScreenMill: A freely available software suite for growth measurement, analysis and visualization of high-throughput screen data*. *BMC Bioinformatics* (Vol. 11). https://doi.org/https://doi.org/10.1186/1471-2105-11-353

Howell, R. S. M., Csikász-Nagy, A., & Thorpe, P. H. (2019). Synthetic physical interactions with the yeast centrosome. *G3: Genes, Genomes, Genetics*, *9*(7), 2183–2194. https://doi.org/10.1534/g3.119.400117
